# Supplementary material for: Genetic Variability of Mineral Content in Different Grain Structures of Bean Cultivars from Mesoamerican and Andean Gene Pools
Source: Plants (Basel). 2021 Jun 19;10(6):1246. doi: 10.3390/plants10061246 (PMC8234366; doi:10.3390/plants10061246)
Supplement: Supplementary file 1 [file plants-10-01246-s001.zip › plants-1206323-supplementary.pdf]

Supplementary Materials

Table S1. Average values obtained for the protein content (Prot) and 100-grain mass (M100) traits evaluated in 10 bean cultivars.

**Cultivar**[1]	**Prot (%)**	**M100 (g)**
IPR Siriri	17.3 b	25.8 d
IPR Juriti	17.2 b	31.3 d
IPR Uirapuru	17.9 b	28.3 d
IPR Gralha	16.7 b	24.8 d
IAPAR 31	14.9 b	26.3 d
IPR Garça	16.9 b	58.3 a
Hooter	19.0 a	49.3 b
BRS Radiant	16.4 b	48.8 b
Jalo Precoce	21.3 a	41.0 c
RedHawk	20.4 a	48.3 b

[1] Median values followed by the same letters show no statistically significant difference, as determined using the Scott–Knott test at the 5% significance level.

Table S2. Mean values of the macronutrients Mg, Ca, K, and P in three grain tissues of 10 bean (*Phaseolus vulgaris* L.) cultivars derived from different centers of origin.

**Cultivar**[1]	**Mg (g kg^{-1} DM)**			**Ca (g kg^{-1} DM)**		
	**Seed coat**	**Cotyledons**	**Embryo**	**Seed coat**	**Cotyledons**	**Embryo**
IPR Siriri	2.37 bA	2.00 aB	1.35 bC	6.51 bA	0.23 aB	0.46 aB
IPR Juriti	2.51 aA	1.87 aB	1.31 bC	6.23 bA	0.20 aB	0.43 aB
IPR Uirapuru	2.32 bA	1.78 aB	1.32 bC	7.07 aA	0.22 aB	0.42 aB
IPR Gralha	2.11 bA	1.89 aA	1.36 aB	6.11 bA	0.17 aB	0.41 aB
IAPAR 31	2.52 aA	1.80 aB	1.26 bC	7.46 aA	0.20 aB	0.38 aB
IPR Garça	2.28 bA	2.05 aA	1.20 aB	5.61 bA	0.20 aB	0.47 aB
Hooter	2.53 aA	1.70 aB	1.18 bC	4.75 cA	0.20 aB	0.42 aB
BRS Radiant	2.50 aA	1.70 aB	1.06 bC	4.59 cA	0.18 aB	0.39 aB
Jalo Precoce	2.32 bA	1.35 bB	1.03 bC	3.94 dA	0.16 bB	0.34 aB
RedHawk	2.50 aA	1.69 aB	1.01 bC	3.98 dA	0.15 bB	0.34 aB
Cultivar	K (g kg^{-1} DM)			P (g kg^{-1} DM)		
	Seed coat	Cotyledons	Embryo	Seed coat	Cotyledons	Embryo
IPR Siriri	0.038 cC	0.168 aA	0.125 aB	0.19 bC	2.96 aB	14.70 aA
IPR Juriti	0.038 cC	0.153 aA	0.120 aB	0.31 aC	2.89 aB	13.67 aa
IPR Uirapuru	0.050 bC	0.138 bA	0.120 aB	0.24 aC	3.10 aB	14.23 aA
IPR Gralha	0.058 bC	0.155 aA	0.133 aB	0.32 aC	2.91 aB	13.68 aA
IAPAR 31	0.078 bC	0.160 aA	0.125 aB	0.25 aC	2.89 aB	14.54 aA
IPR Garça	0.080 aC	0.160 aA	0.130 aB	0.21 aC	2.64 aB	13.05 aA
Hooter	0.048 bC	0.160 aA	0.135 aB	0.18 bC	2.80 aB	14.95 aA
BRS Radiant	0.035 cC	0.143 bA	0.125 aB	0.19 bC	2.49 aB	15.18 aA
Jalo Precoce	0.043 cC	0.160 aA	0.120 aB	0.18 bC	3.13 aB	13.90 aA
RedHawk	0.040 cC	0.168 aA	0.118 aB	0.19 bC	2.90 aB	14.72 aA

[1] Median values followed by the same uppercase letters in rows and lowercase letters in columns show no statistically significant difference, as determined using the Scott–Knott test at the 5% significance level.

Table S3. Means values for contents of the micronutrients Mn, S, Cu, B, Fe, and Zn evaluated in three grain tissues of 10 bean (*Phaseolus vulgaris* L.) cultivars derived from different centers of origin.

Cultivar[1]	Mn (mg kg^{-1} DM)			S (g kg^{-1} DM)			Cu (mg kg^{-1} DM)		
	Seed coat	Cotyledons	Embryo	Seed coat	Cotyledons	Embryo	Seed coat	Cotyledons	Embryo
IPR Siriri	3.4 aB	18.6 aA	16.8 aA	0.79 aB	3.02 aA	1.15 bB	5.1 aC	9.2 aB	19.0 bA
IPR Juriti	3.6 aB	13.2 bA	15.1 aA	0.64 aB	2.85 aA	1.10 bB	4.1 bC	10.0 aB	19.1 bA
IPR Uirapuru	4.7 aB	17.7 aA	17.2 aA	0.84 aB	3.12 aA	1.13 bB	5.4 aC	10.4 aB	20.6 aA
IPR Gralha	4.1 aB	14.6 bA	16.1 aA	1.00 aB	2.86 aA	1.16 bB	6.1 bC	11.2 aB	22.3 aA
IAPAR 31	4.9 aB	17.6 aA	16.9 aA	0.97 aB	2.88 aA	1.18 bB	5.1 aC	7.5 aB	15.2 dA
IPR Garça	4.8 aB	14.4 bA	16.0 aA	0.74 aB	2.65 aA	2.60 aA	6.7 aB	8.4 aB	17.4 cA
Hooter	5.4 aB	13.7 bA	15.9 aA	0.56 aB	2.89 aA	1.18 bB	6.4 bC	8.9 aB	18.7 bA
BRS Radiant	3.7 aB	12.5 bA	15.3 aA	0.69 aB	2.51 aA	1.12 bB	6.0 bC	9.1 aB	19.5 bA
Jalo Precoce	4.4 aC	12.3 bB	16.1 aA	0.57 aB	3.22 aA	1.10 bB	6.1 bC	10.1 aB	18.7 bA
RedHawk	4.2 aC	11.0 bB	14.8 aA	0.69 aB	2.85 aA	1.16 bB	5.9 bC	9.6 aB	21.0 aA

Cultivar	B (mg kg^{-1} DM)			Fe (mg kg^{-1} DM)			Zn (mg kg^{-1} DM)		
	Seed coat	Cotyledons	Embryo	Seed coat	Cotyledons	Embryo	Seed coat	Cotyledons	Embryo
IPR Siriri	9.3 aB	6.8 aB	36.3 aA	75.3 dB	37.7 aC	115.2 bA	26.5 bB	24.2 aB	80.7 aA
IPR Juriti	10.8 aB	6.9 aB	23.5 bA	100.6 cA	39.6 aB	110.7 bA	28.2 bB	23.6 aB	81.1 aA
IPR Uirapuru	11.4 aB	7.2 aB	30.3 bA	119.5 bA	39.6 aB	131.4 aA	32.8 aB	25.8 aB	75.2 bA
IPR Gralha	11.4 aB	6.5 aB	29.8 bA	144.2 aA	33.6 bC	117.7 bB	23.3 bB	24.9 aB	80.2 aA
IAPAR 31	10.0 aB	6.2 aB	27.4 bA	81.2 dB	34.9 bC	104.7 bA	34.1 aB	23.5 aB	85.5 aA
IPR Garça	12.6 aB	6.5 aB	27.1 bA	64.1 eB	35.9 bC	107.3 bA	12.1 cC	18.0 bC	73.1 bA
Hooter	11.3 aB	6.3 aB	35.8 aA	47.9 eB	41.6 aB	107.4 bA	12.2 cC	20.6 bC	68.5 cA
BRS Radiant	11.5 aB	6.0 aB	36.0 aA	56.7 eB	35.7 bC	110.1 bA	10.4 cC	17.8 bC	66.2 cA
Jalo Precoce	11.6 aB	6.0 aB	32.7 aA	61.3 eB	42.1 bC	128.4 aA	11.2 cC	23.6 aB	63.6 cA
RedHawk	11.2 aB	6.3 aB	33.2 aA	60.5 eB	34.2 bC	95.2 bA	12.9 cC	21.0 aC	65.3 cA

[1] Median values followed by the same uppercase letters in rows and lowercase letters in columns show no statistically significant difference, as determined using the Scott–Knott test at the 5% significance level.

Table S4. Mean percentages of the biomass of the seed coat, cotyledons, and embryonic axis relative to the total grain biomass of 10 bean cultivars (*Phaseolus vulgaris* L.) cultivars.

Cultivar	Average percentage dry biomass		
	Seed coat	Cotyledons	Embryo
IPR Siriri	7.8	90.8	1.4
IPR Juriti	8.0	90.6	1.4
IPR Uirapuru	8.5	90.1	1.4
IPR Gralha	8.9	89.5	1.6
IAPAR 31	7.7	90.9	1.4
IPR Garça	8.2	90.7	1.1
Hooter	9.2	89.5	1.3
BRS Radiant	8.0	90.9	1.1
Jalo Precoce	8.5	90.4	1.1
RedHawk	8.7	90.2	1.1
Average	8.3	90.4	1.3

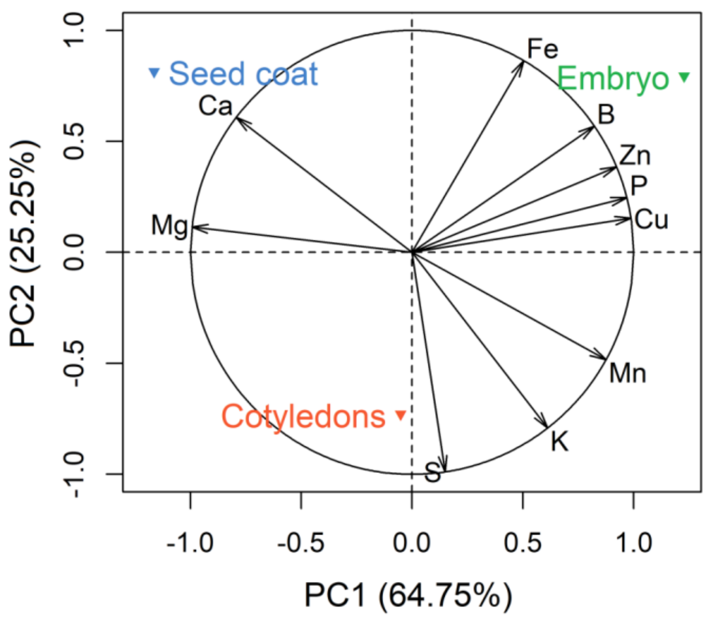

Figure S1. Principal component analysis of the contents of 10 minerals in the seed coat, cotyledons, and embryonic axis of the grains of 10 bean cultivars (*Phaseolus vulgaris* L.) cultivars.
